# Esterase Active in Polar Organic Solvents from the Yeast *Pseudozyma* sp. NII 08165

**DOI:** 10.1155/2014/494682

**Published:** 2014-04-03

**Authors:** Deepthy Alex, Anju Shainu, Ashok Pandey, Rajeev K. Sukumaran

**Affiliations:** ^1^Biotechnology Division, CSIR-National Institute for Interdisciplinary Science and Technology, Thiruvananthapuram 19, India; ^2^Mar Ivanios College, Thiruvananthapuram 695015, India

## Abstract

Esterases/lipases active in water miscible solvents are highly desired in biocatalysis where substrate solubility is limited and also when the solvent is desired as an acyl acceptor in transesterification reactions, as with the case of biodiesel production. We have isolated an esterase from the glycolipid producing yeast-*Pseudozyma* sp. NII 08165 which in its crude form was alkali active, thermo stable, halo tolerant and also capable of acting in presence of high methanol concentration. The crude enzyme which maintained 90% of its original activity after being treated at 70°C was purified and the properties were characterized. The partially purified esterase preparation had temperature and pH optima of 60°C and 8.0 respectively. The enzyme retained almost complete activity in presence of 25% methanol and 80% activity in the same strength of ethanol. Conditions of enzyme production were optimized, which lead to 9 fold increase in the esterase yield. One of the isoforms of the enzyme *LIP1* was purified to homogeneity and characterized. Purified *LIP1* had a *K*_*m*_ and *V*_max_ of 0.01 and 1.12, respectively. The purified esterase lost its thermo and halo tolerance but interestingly, retained 97% activity in methanol.

## 1. Introduction

Lipases (triacylglycerol acylhydrolase, EC 3.1.1.3) are part of the family of hydrolases that act on carboxylic ester bonds. Their natural substrates are triacylglycerols and under natural conditions they catalyze the hydrolysis of ester bonds at the interface between an insoluble substrate phase and the aqueous phase in which the enzyme is dissolved [1]. A reverse reaction where fatty acids and glycerol are esterified to form glycerides is preferred in the absence of water. Lipases/esterases find applications in a variety of fields including food and dairy industry, detergent, pharmaceuticals, agrochemical, and oleochemical industries [2, 3]. They also serve as potential biocatalysts in several biotransformations of industrial importance.

Lipases/esterases with novel properties are always in demand due to the large number of synthetic reactions which these enzymes can possibly catalyze and for which enzymatic routes are currently not available [4]. Novel specificities, tolerance to extremes of pH, temperature, and/or salt tolerance are features often desired in esterases. Biocatalysis in organic solvents are advantageous due to several reasons including the ability to perform reactions restricted kinetically or thermodynamically in water, increased solubility of certain substrates in organic solvents, suppression of undesirable side reactions, possibility to control or modify the enzyme selectivity, and increased thermal stability in organic solvents [5–7]. It has been observed that the enzyme activity and stability are higher in hydrophobic solvents compared to hydrophilic solvents since the latter can strip the water required for activity [7]. At the same time, there are reactions which perform better in polar organic solvents and hence the lipases/esterases that work in such systems are also highly desired in the industry [8].* Pseudozyma* sp. isolated at the biotechnology division of CSIR-NIIST was found to produce esterase activity which was sustained at higher temperature and under high salt concentration. The enzyme was more active under alkaline pH and it retained activity in presence of polar organic solvents like ethanol and methanol. The present work explored the possibility of using this novel isolate as a source of esterase active in polar organic solvents and for characterizing the enzyme for properties that are desirable for industrial esterases.

## 2. Materials and Methods

### 2.1. Strain and Cultivation Conditions

The esterase-producing strain was isolated through an aerial sampling and was identified by molecular methods as* Pseudozyma *sp. [8]. The culture designated as NII 08165 was maintained on potato-dextrose agar (PDA) slants and was subcultured fortnightly. Growth and enzyme production were carried out in a basal mineral salts medium (pH 6.00) containing in gl^−1^(NH_4_)_2_SO_4_-2.0, KH_2_PO_4_-5.0, MgSO_4_·7H_2_O-2.0, Peptone-1.0, Glucose-10, with up to 1% olive oil supplementation in the case of enzyme production medium. The seed culture was grown in the basal medium at 30°C and with 200 rpm agitation in a shaker incubator till it reached 1.00 OD. Enzyme production medium was inoculated at up to 2.0% v/v level and the cultivation was carried out at 30°C for 4 days with 200 rpm agitation. At the end of the cultivation, the culture was centrifuged at 10,000 rpm and 4°C for 15 min and the culture supernatant was used as the crude enzyme preparation.

### 2.2. Enzyme Assay

Esterase activity was measured as the ability to hydrolyze para-nitrophenyl palmitate (pNPP) by a modified method of Gupta et al. [9]. Appropriate volume enzyme was incubated with 1 mM solution of pNPP in buffer A (50 mM Tris, pH 8, containing 50 mM NaCl, 0.4% Triton X-100 and 0.1% Gum Arabic). The reaction mixture was incubated at 50°C for 30 min and absorbance readings were taken at 405 nm in a UV-Visible spectrophotometer. Standards were run with appropriate concentrations of para-nitrophenol (pNP). One unit of esterase activity was defined as the amount of enzyme liberating 1 *μ*M of pNP per millilitre per minute.

### 2.3. Enhancing Esterase Production by Process Optimization

Optimization of medium components and process parameters for esterase production was performed in two stages. Initially, 7 variables were screened using a fractional factorial design to identify the parameters which significantly influenced enzyme production and in the second stage the levels of these parameters were optimized using a response surface design. The initial screening of parameters was performed following a Plackett & Burman experimental design [10] with 7 variables at two levels in a total of 8 experimental runs. The design matrix for the experiment was generated with the DOE software-Design Expert (Stat-Ease Corp, Minneapolis, USA) and is given in Table 1.

A factorial model was fitted for the main effects using Design Expert software (Statease Corp., USA). The effects of individual parameters on esterase production were calculated by the following equation:

(1) $$\begin{array}{c} \varepsilon =\frac{\left(\sum_{}^{}\mu_{+}-\sum_{}^{}\mu_{-}\right)}{n}, \end{array}$$

where *ε* is the effect of parameter under study and “*μ*_+_” and “*μ*_−_” are responses (esterase activities) of trials at which the parameter was at its higher and lower levels, respectively, and “*n*” is the total number of trials. Analysis of variance (ANOVA) was performed on the data to determine the significance of fitted model and to test the significance of the effect of individual parameters on esterase production. The most significant parameters affecting esterase production were identified and were optimized following a response surface Box Behnken design [11] where the effect of the significant variables was studied at three different levels (Table 2). The behaviour of the system was modeled by a second-order polynomial equation. The model equation used for the analysis is given below:

(2) $$\begin{array}{c} Y=\beta_{0}+\overset{n}{\underset{i=1}{\sum }}\beta_{i}X_{i}+\overset{n}{\underset{i=1}{\sum }}\beta_{ii}X_{i}^{2}+\overset{n}{\underset{i=1\;}{\sum }}\overset{n}{\underset{j=1}{\sum }}\beta_{ij}X_{i}X_{j}, \end{array}$$

where *Y* is the predicted response; *β*_0_ is the offset term; *β*_*i*_ is the linear effect; *β*_*ii*_ is the squared effect; *β*_*ij*_ is the interaction effect; and *X*_*i*_ and *X*_*j*_ are coded terms for independent variables under study. Regression analysis and estimation of the coefficients were performed using Design Expert. Effect estimates, the three-dimensional response surfaces and contour plots, were generated using Design Expert and Microsoft Excel. The ideal levels and combinations of parameters were identified by optimization functions in the Design Expert software and experiments were run at these levels for validation of the model. Enzyme production for purification and characterization studies was done under the optimized conditions.

### 2.4. Studies on the Properties of the Enzyme

Crude enzyme was concentrated by ultrafiltration using a 10 kD cut-off membrane and was subjected to ammonium sulfate precipitation using 60% (NH_4_)_2_SO_4_. The pellets were dissolved in a minimal volume of 100 mM Tris buffer (pH 8.0) and were dialyzed against excess volume of 100 mM Tris buffer (pH 8.0) for salt removal. The concentrated, partially purified enzyme sample as above was used for studies on the properties of the esterase. The enzyme was tested for tolerance to temperature, solvents, and salt, and its stability at elevated temperatures was also studied.

#### 2.4.1. Temperature Tolerance and Temperature Stability

The temperature tolerance of esterase was studied by incubating the enzyme samples at 37, 50, 60, and 70°C for 30 min. The activities obtained at different incubation temperature were compared against the enzyme activity at 37°C which was taken as the control and was expressed as percentage of activity in comparison to the control. Temperature stability of the enzyme was studied by preincubating the enzyme at different temperatures (40, 50, 60, and 70°C) for 1 h followed by assay of the enzyme at 37°C. The activities obtained were compared to the control enzyme incubated at room temp (30 ± 2°C) and assayed at 37°C.

#### 2.4.2. pH Tolerance

pH tolerance of esterase was evaluated by performing the assays in different buffers: Na-acetate (pH 2–5), Na-Phosphate (pH 6–8), and Tris buffer (pH 8–10). The activity and stability at different pH were tested by preincubating the enzyme in different buffers for 1 h followed by assays for enzyme activity in the respective buffers at 37°C.

#### 2.4.3. Solvent Tolerance

The enzyme was assayed in presence of 25% and 50% of the polar water miscible organic solvents—isopropanol, acetonitrile, ethanol, or methanol. The solvents were incorporated at appropriate concentrations into the assay mixture and the assays were performed at 37°C.

#### 2.4.4. Halo Tolerance

The salt tolerance of the enzyme preparation was tested by performing the esterase assays in presence of varying concentrations of sodium chloride. NaCl was added to the assay mixture to obtain final concentration in the range 50 mM–2.0 M and assay was performed as above.

### 2.5. Purification and Characterization of Esterase Isoform

Esterase production was carried out under optimised media and culture conditions in shake flasks. Cell-free supernatant (1950 mL) obtained after centrifugation of cell homogenate at 4°C and 10,000 rpm for 15 min was concentrated by tangential flow ultrafiltration using a 50 kD cut-off membrane. Retentate obtained after ultrafiltration was further purified by ammonium sulfate fractionation and hydrophobic interaction chromatography (HIC). The fraction obtained from the 60% ammonium sulfate precipitation (17.5 mL) showing maximum esterase activity was dialysed against 20 mM HEPES buffer (pH 8.0) containing 1.0 M (NH_4_)_2_SO_4_. This was loaded onto Butyl Sepharose column (1.5 × 30 cm) previously equilibrated with the same buffer to maintain similar salt conditions. The column was washed until all unbound protein was eluted. Elution of bound proteins was done with a 1.0–0.0 M linear gradient of (NH_4_)_2_SO_4_ in 20 mM HEPES buffer (pH 8.0) at a flow rate of 1 mL min^−1^. Fractions of 2 mL were collected and assayed for esterase activity. The active fractions were pooled and dialysed against 20 mM HEPES buffer to remove salt.

#### 2.5.1. Enzyme Characterization

The HIC purified enzyme was run on SDS PAGE and its molecular weight was determined according to Laemmli [12]. Enzyme kinetic studies were performed and the Michaelis-Menten curve fitting and the determination of *V*_max⁡_ and *K*_*m*_ were performed using GraphPad Prism software (GraphPad Software Inc., La Jolla, CA, USA). The kinetic parameters of the purified esterase were determined at the optimum temperature of 60°C and pH 8, using 0.05–0.7 mM pNPP as substrate. The purified esterase isoform was analyzed for its solvent, temperature, and salt tolerance as described above.

### 2.6. Experiment Analyses

All experiments were performed in triplicates unless otherwise indicated and numerical data were statistically analyzed using Microsoft Excel. DOE data were analyzed using Design expert (Stat Ease Corp, USA). Graphical representations of data were generated using either SigmaPlot (Systat Software, USA) or Microsoft Excel and statistically significant differences in enzyme activity between experiments were denoted by asterisks (∗).

## 3. Results

### 3.1. Screening of Parameters Affecting Esterase Production

The responses obtained for the screening experiment based on the Plackett and Burman design are given in Table 1. Effect estimate of the various parameters indicated that pH, Inoculum concentration, and incubation time had the highest effects. The results were analyzed statistically and a first-order polynomial equation was derived to represent esterase production as a function of the independent variables:

(3) $$\begin{array}{c} Y=983+480X_{4}-795X_{5}-474X_{6}, \end{array}$$

where *Y*-Esterase Activity, *X*_4_-inoculum concentration, *X*_5_-pH, *X*_6_-Incubation time. Adequacy of the model was tested and the parameters with significant effects were identified by Fisher's test for analysis of variance (ANOVA). The model had an *F* value of 12.93 and the Prob > *F* value of 0.016 which indicated that the model was significant. The parameters with significant effects were inoculum concentration, pH of the medium, and incubation time with confidence levels above 95% (Prob > *F* ≤ 0.05).

### 3.2. Optimization of Critical Parameters Identified by Factorial Design

The Box Behnken experiment design matrix and the experimental and predicted responses obtained for esterase production by* Pseudozyma *are shown in Table 2. The data was analyzed by multiple regression analysis and a second-order polynomial equation was derived to represent the esterase production as a function of the independent variables tested:

(4) Y=6729+1257X1+2874X2−322X3−2637X12−1079X22−1786X32+1182X1X2−649X1X3+431X1X2,

where *Y* = predicted response (*e* yield), *X*_1_, *X*_2_, and *X*_3_ are coded values of inoculum concentration, pH, and Incubation time, respectively.

Testing of the model was performed by Fisher's statistical test for the analysis of variance (ANOVA) using Design Expert software. ANOVA of the quadratic regression model suggested that the model was significant with a computed *F* value of 7.13 and a *P* > *F* of 0.0084. The value of multiple correlation coefficient (*R*) was 0.9495 indicating good correlation. The model terms which had a *P* value <0.05 indicated their significance and in this case *X*_1_ *X*_2_, *X*_1_^2^, and *X*_3_^2^ were found to be significant model terms. There was no significant interaction between the parameters.

**Figure 1:**
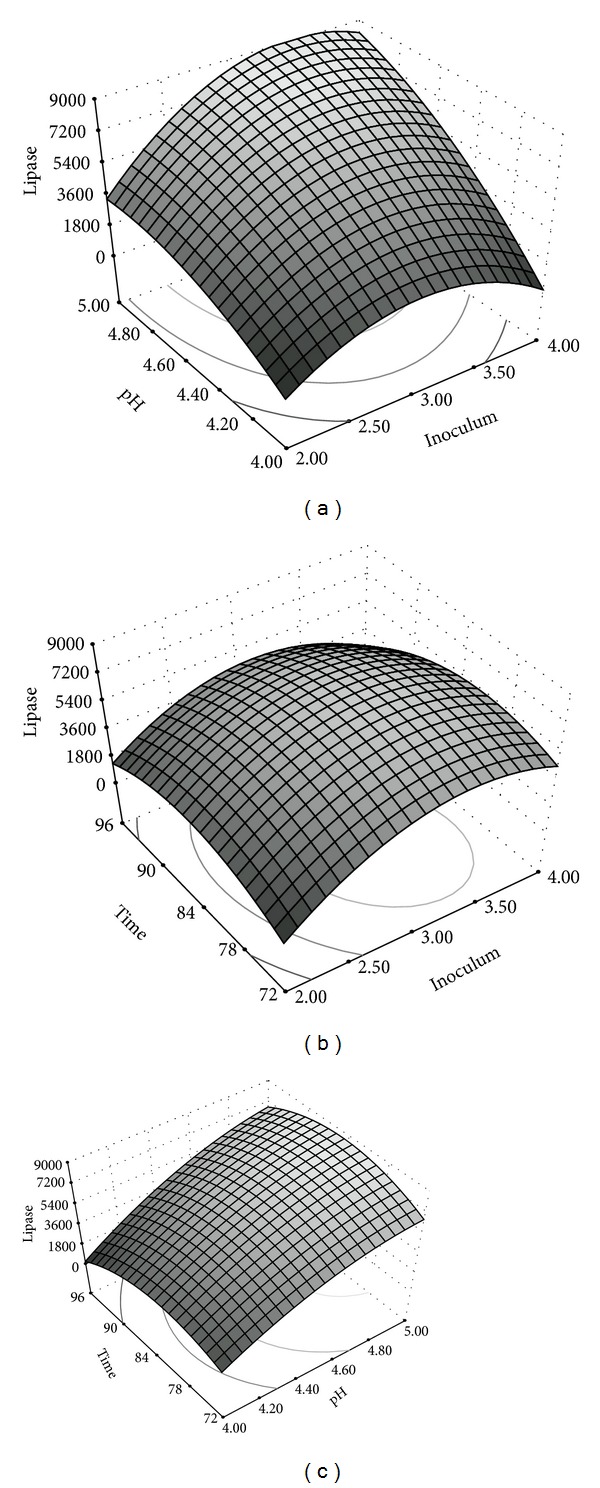
Response surface plots showing interaction of parameters affecting esterase production by* Pseudozyma* sp. NII 08165. (a) Interaction between inoculum concentration and pH. (b) Interaction between inoculum concentration and incubation time. (c) Interaction between pH and incubation time.

Figures 1(a), 1(b), and 1(c) represent the response surfaces for the interaction effects of inoculum concentration & pH, inoculum concentration and incubation time, and pH and incubation time, respectively. The highest esterase yield was obtained with an inoculum concentration of ~3.5% and at a medium pH of 5 (Figure 1(a)). At lower medium pH, higher esterase activities were obtained with lower inoculum concentration, whereas at higher medium pH an increased inoculum concentration was necessary to obtain similar enzyme yield. At lower pH, the cells could be stressed resulting in a higher production and/or secretion of the enzyme.

Irrespective of the inoculum concentration, the maximal esterase yield was obtained at an incubation time of 80–84 h (Figure 1(b)). The inoculum concentration which gave maximal enzyme production was independent of the incubation period and the optimal level was between 3.0 and 3.5%. The time required for obtaining maximal esterase yield was also influenced by the pH of the medium. At lower pH, the esterase yield increased with increase in incubation time and peaked at around 78–80 h, while, at higher pH, the peak production was observed between 80 and 84 h (Figure 1(c)). However, it may be noted that there was not much difference in the incubation time needed for peak production and the interaction between these parameters was not so pronounced. The highest esterase activity obtained in this study was 8113 U/mL after the two-stage statistical design mediated process optimization. He and Tan [13] reported similar high titres of lipase activity in* Candida* sp., where the lipase yield increased 1.54-fold to 6230 U/mL after optimising culture medium in shake flask system.

### 3.3. Studies on the Properties of Partially Purified Enzyme

#### 3.3.1. Temperature Tolerance and Temperature Stability

**Figure 2:**
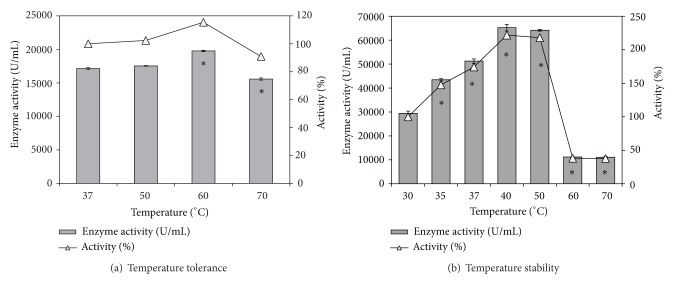
Temperature tolerance and stability of partially purified esterase preparation from* Pseudozyma* sp. NII 08165. Bars represent enzyme activity and open triangles show the % activity retention compared to control. *Enzyme activity values significantly different from control.

Thermostable lipases/esterases are always in industrial demand because of the high melting point of several lipid substrates participating in the process, and also since a higher reaction rate can be achieved at elevated temperatures [14, 15]. The partially purified esterase from* Pseudozyma *sp. NII 08165 showed maximal activity at 60°C and more than 90% of the activity was retained at 70°C which may qualify it as a thermotolerant esterase (Figure 2(a)). The results for the temperature stability study are given in Figure 2(b). Preincubation of enzyme at temperatures ranging from 35 to 50°C actually increased the activity of the enzyme preparation in comparison to the control which was preincubated at 30°C. Incubation of the enzyme at either 40°C or 50°C resulted in more than twofold increase (~220%) in enzyme activity indicating the stability of the enzyme at these temperatures. Nevertheless, the enzyme lost its activity significantly after 1 h incubation at either 60°C or 70°C. In the latter case the activity retained was ~40% of the control which was incubated at 30°C.

#### 3.3.2. pH Tolerance

**Figure 3:**
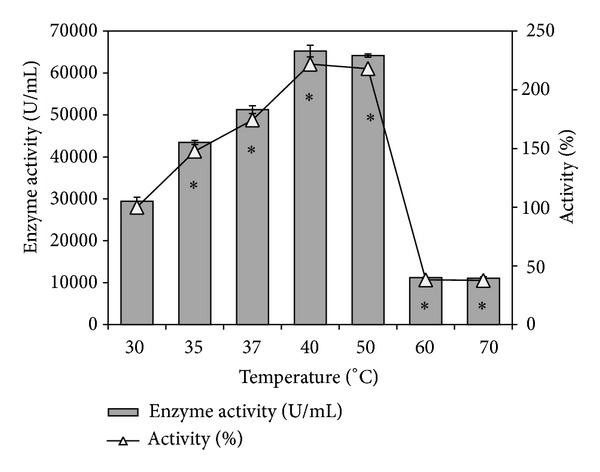
Activity of the partially purified* Pseudozyma *esterase at different pH. *Enzyme activity values are significantly different from control.

A slightly alkaline pH supported maximal esterase activity for the* Pseudozyma* enzyme (Figure 3). Maximal activity was obtained at pH 8.0 which was ~140% higher than the activity for control (pH 7.2). While the enzyme retained 70% of its activity at neutral pH, the activity retentions for pH 9.0 and 10.0 were only 46% and 34%. The enzyme was not active in the acidic pH, indicating a confined operating range. The optimal pH range for activity was 7-8, with pH 8.0 supporting maximal activity. However, the ability of the enzyme to act at pH 9.0 and 10.0 positions itself with a definite advantage for use in applications that require alkaline conditions.

#### 3.3.3. Solvent Tolerance

**Figure 4:**
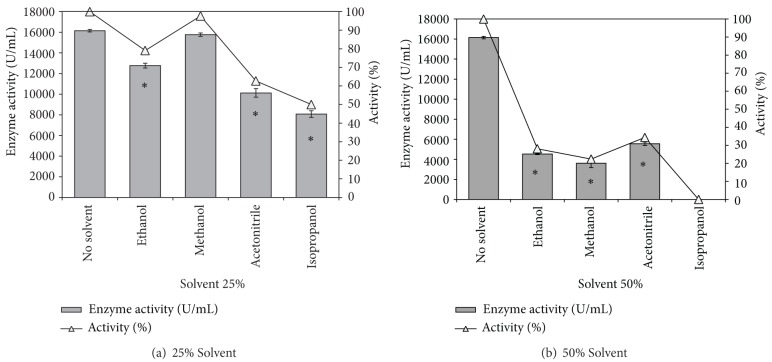
Solvent tolerance of partially purified esterase from* Pseudozyma* sp. NII 08165. Bars represent enzyme activity and open triangles shows the % activity retention compared to control. *Enzyme activity values significantly different from control.

Lipases/esterases are more susceptible to inactivation in presence of water miscible organic solvents and tolerance to polar organic solvents is a highly desired property in lipases/esterases due to a range of reactions requiring such solvents [8, 16]. The* Pseudozyma *esterase was active in 25% concentration of all the tested organic solvents. In 25% methanol 97% of the activity was retained compared to the control (solvent free) while 80% of activity was retained in presence of the same concentration of ethanol (Figure 4(a)). The tolerance to acetonitrile and isopropanol was 63% and 50%, respectively. Though there are reports of esterases with enhanced activity or activity retention in polar organic solvents [17, 18], the* Pseudozyma *esterase differed from them in the high titres of enzyme activity obtained. At 50% concentration, the enzyme lost most of its activity in all the tested solvents. The maximum activity retention at this concentration was 34% for acetonitrile (Figure 4(b)). 75–80% activity was lost in ethanol and methanol, while the enzyme completely lost its activity in 50% isopropanol. Nevertheless, it may be observed that the enzyme is active in presence of 25% of the major polar organic solvents used in synthesis reactions.

#### 3.3.4. Halotolerance

**Figure 5:**
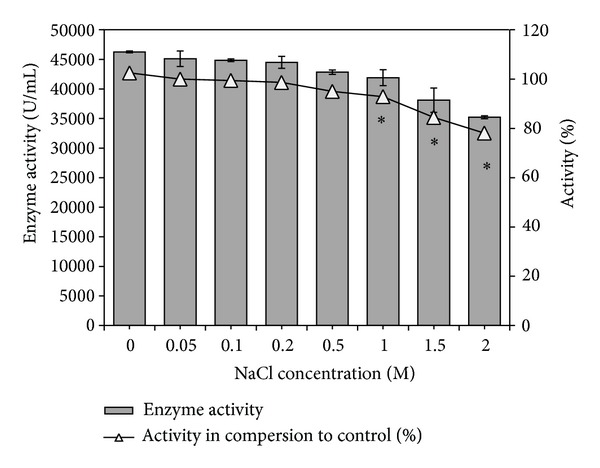
Halo-tolerance of partially purified* Pseudozyma* esterase. *Enzyme activity values significantly different from control.

The esterase from* Pseudozyma* sp. seemed to be halotolerant as it was active in buffer containing 0.05–2.0 M NaCl (Figure 5). While esterases from halophilic microbes are in most cases active only at high salt concentrations, the salt tolerant esterases from mesophiles can act both in the absence of NaCl and in a range of NaCl concentrations. The* Pseudozyma *esterase may be considered under the latter category which might be advantageous in several applications like waste water and oil spill management [3, 19].

### 3.4. Purification of Esterase

**Figure 6:**
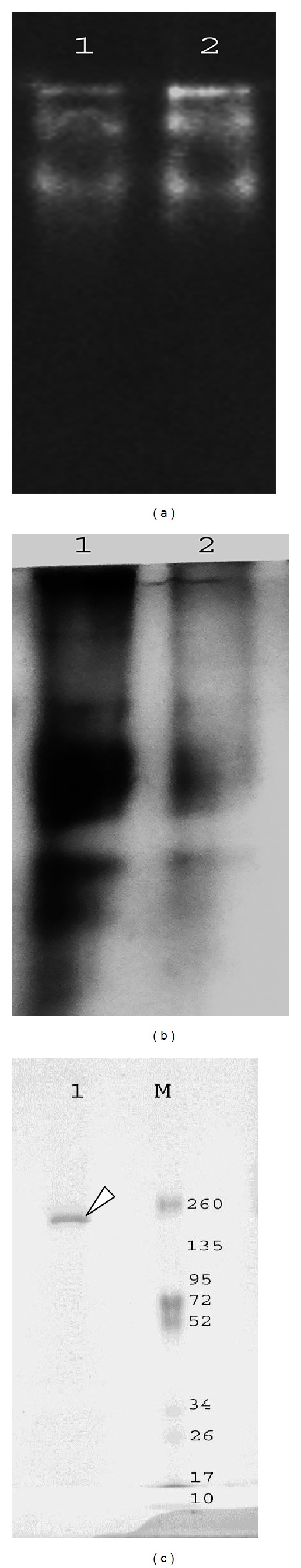
Purification of* LIP1* esterase. (a) Zymogram analysis of* Pseudozyma* esterases. Lanes: 1: crude enzyme, 2: ammonium sulfate fraction. (b) Native PAGE analysis of esterases. Lanes: 1: crude enzyme, 2: ammonium sulfate fraction. (c) SDS PAGE analysis of purified* LIP1*, Lane 1: purified* LIP1* esterase, Lane M: SDS broad range protein ladder (Spectra, Thermo Scientific). The 175 kD* LIP1* esterase protein band is indicated by arrow head.

Crude esterase was concentrated and fractionated by ultrafiltration through a 50 kD cut-off membrane to obtain an 8.5-fold purification followed by ammonium sulfate fractionation. Maximal esterase activity was detected in the 60% (w/v) ammonium sulfate fraction, though the other fractions also contained appreciable amount of esterase activity. An activity staining of the partially purified esterase using 4-methyl umbelliferyl palmitate [20] indicated the presence of multiple isoforms esterase (Figure 6). The enzyme was further purified to ~82-fold through hydrophobic interaction chromatography (Table 3).

The purified esterase on SDS-PAGE followed by silver staining showed a single band of molecular weight 175 kDa (Figure 6(c)). Due to the surface hydrophobic patches on esterases, ammonium sulfate precipitation followed by HIC can be considered as an effective two-step purification strategy for esterases [21].

**Figure 7:**
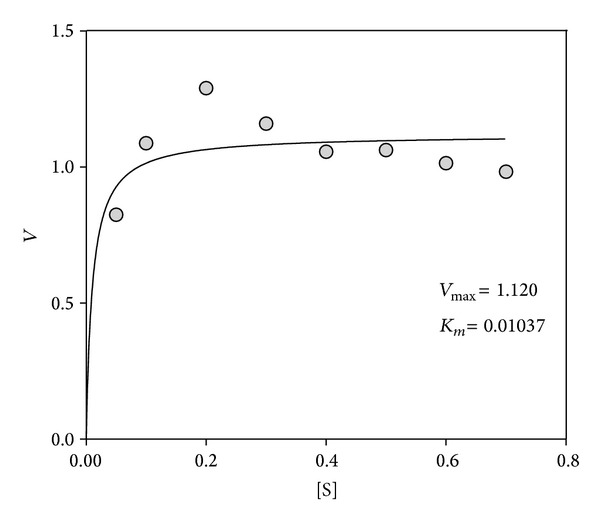
Michaelis Menten Curve for* Pseudozyma* sp.* LIP1.*

Kinetic parameters of the purified esterase were determined in a normal assay mixture with pH 8, incubation temperature of 60°C and using a substrate (pNPP) concentration range of 0.05–0.7 mM. Michaelis-Menten kinetics was fitted and the *K*_*m*_ and *V*_max⁡_ were determined to be 0.01 mM and 1.12 mM min^−1^ (Figure 7). The purified esterase isoform was designated as* LIP1*.

### 3.5. Properties of the Purified* LIP1*

**Figure 8:**
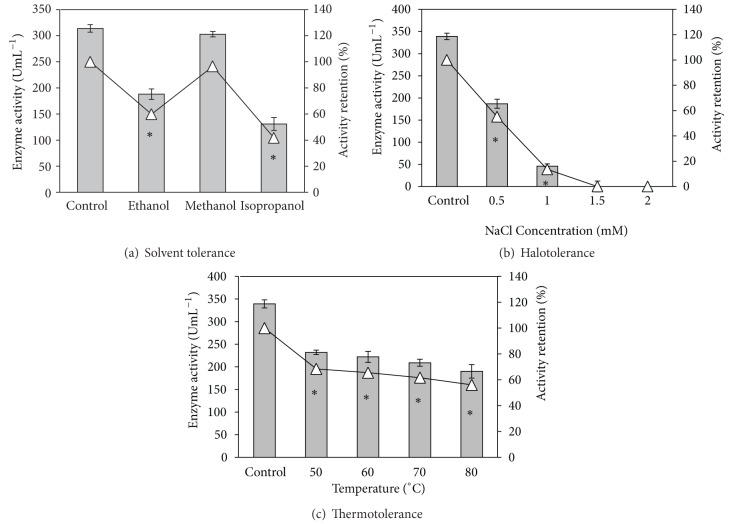
Properties of purified* LIP1* esterase. *Enzyme activity values are significantly different from control.

The purified* LIP1* showed only 60% and 42% activity retention in ethanol and isopropanol whereas the partially purified esterase showed 80% and 50% activity retention, respectively, in these polar organic solvents. It may be noted that there were more than one esterase isoforms in the crude esterase and the isoforms other than* LIP1* could have contributed to the higher solvent tolerance of the partially purified enzyme. Interestingly, the activity retention for purified* LIP1* in 25% methanol (97%) was identical to the partially purified enzyme (Figure 8(a)).

The halo tolerance of purified esterase decreased sharply compared to the crude enzyme (Figure 8(b)). While the partially purified esterase showed more than 80% activity retention in 1.5–2.0 M NaCl concentration, there was a complete loss of activity for* LIP1*, even at 1.5 M salt concentration indicating that the halotolerance exhibited by the partially purified enzyme was contributed by the isoforms other than* LIP1*. It is known that there is a correlation between the salt concentration and enzyme activity which was proposed to be due to the changes in protein functional groups hydration which in turn bring about changes in the free energy of activation [22]. The temperature tolerance for purified* LIP1* also decreased significantly compared to the partially purified enzyme. While the partially purified esterase retained 90% activity after treating the enzyme at 70°C for 1 h, purified esterase could retain only 62% of its original activity under the same conditions (Figure 8(c)).

## 4. Discussion

The* Pseudozyma* isolate was obtained through aerial sampling in triolein-rhodamine agar plates made for screening of esterase producers. The colonies of this culture brightly fluoresced in UV light indicating esterase production and initial trials showed high titres of enzyme activity. The culture was also found to produce mannosylerythritol lipids [8], and hence it was decided to look further into enzyme production by this organism and study the properties of the enzyme.

The medium and conditions were optimized in a two-stage process employing fractional factorial and response surface designs sequentially to maximize enzyme production. The optimization of process variables resulted in an almost 9-fold (903 U/mL to 8113 U/mL) improvement in enzyme production. This is one of the highest esterase activities reported and shows the potential for commercial exploitation of the organism.

The studies done on the properties of the partially purified esterase from* Pseudozyma* sp. NII 08165 indicated the great potential of this enzyme. The partially purified esterase was active at 60°C and had an enhanced activity after incubating at 50°C for 1 h. The enzyme may therefore be called thermotolerant. Besides temperature tolerance, the enzyme preparation also exhibited other interesting features like an alkaline pH range for optimal activity, tolerance to water miscible organic solvents, and the ability to act in a wide range of salt concentrations. While there could be enzymes which have better performance in any of these aspects, the occurrence of all these properties in a single enzyme preparation is interesting.

One of the isoforms of this enzyme,* LIP1*, was purified to homogeneity and the purified isoform retained 97% of its activity in 25% methanol, a polar organic solvent which is the most common acyl acceptor used in the transesterification reaction for biodiesel production. Esterases stable in polar organic solvents are advantageous since the water miscible solvent and water can act together as a homogenous cosolvent system and possibly help in reactions involving substrates that are otherwise insoluble in water [23]. It was also reported that water miscible organic solvent systems enhanced hydrolysis of hydrophobic substrates compared to the hydrophilic substrates and such solvents systems may help to modulate the esterase activity [24]. There are several efforts worldwide to enhance the stability and activity of esterases in polar organic solvents ranging from prior treatment with such solvents [25] to structural modifications of the protein [23]. An esterase naturally tolerant to polar organic solvent assumes importance in this context and the* Pseudozyma* sp. esterase with its added advantage of high enzyme titres makes it a potent candidate for industrial applications both in hydrolysis and synthesis. Even though the partially purified esterase preparation exhibited temperature and halotolerance, these properties were not observed in the purified* LIP1* indicating that those properties might have been contributed by other esterases in the preparation. Studies on purifying these isoforms are ongoing.

## 5. Conclusions

Esterase active in methanol -a polar organic solvent was isolated from a novel yeast* Pseudozyma* sp. NII 08165. Crude enzyme preparations from the yeast showed a range of important properties like temperature, pH, solvent, and halotolerance, while the purified* LIP1* protein retained only the solvent tolerance in methanol. Naturally, solvent tolerant enzyme is highly advantageous and the enzyme with its important properties and very high yields can find applications in several industries including synthesis, biodiesel production, and detergents.

## Figures and Tables

**Table 1:** Plackett and Burman design matrix for screening for process variables and the responses obtained for different trials.

Std. order	Glucose (% w/v)	Peptone (% w/v)	Olive oil (% v/v)	Inoculum (% v/v)	pH	Time (h)	(NH_4_)_2_SO_4_ (% w/v)	Esterase (UmL^−1^)
1	0.5	0.6	0.1	2	6.5	120	0.4	169
2	1.0	0.6	0.1	1	4.0	120	1.0	1490
3	0.5	1.2	0.1	1	6.5	96	1.0	142
4	1.0	1.2	0.1	2	4.0	96	0.4	2686
5	0.5	0.6	0.3	2	4.0	96	1.0	2777
6	1.0	0.6	0.3	1	6.5	96	0.4	224
7	0.5	1.2	0.3	1	4.0	120	0.4	158
8	1.0	1.2	0.3	2	6.5	120	1.0	219

**Table 2:** Box Behnken design matrix and responses obtained for the response surface optimization of esterase production by *Pseudozyma* sp.

Std. order	Inoculum conc. (% v/v)	pH	Incubation time (h)	Esterase activity (UmL^−1^)	
				Obtained	Predicted
1	2	4.0	84	419	63
2	4	4.0	84	764	213
3	2	5.0	84	2897	3447
4	4	5.0	84	7970	8325
5	2	4.5	72	1633	721
6	4	4.5	72	5251	4533
7	2	4.5	96	658	1375
8	4	4.5	96	1679	2591
9	3	4.0	72	474	1742
10	3	5.0	72	6267	6629
11	3	4.0	96	598	237
12	3	5.0	96	8113	6846
13	3	4.5	84	7601	6729
14	3	4.5	84	7806	6729
15	3	4.5	84	7652	6729
16	3	4.5	84	4472	6729
17	3	4.5	84	6113	6729

**Table 3:** Summary of purification of esterase from *Pseudozyma* sp.

	Volume (mL)	Esterase activity (U)	Total activity (U)	Protein content (mg mL^−1^)	Total protein (mg)	Specific activity (Umg^−1^)	Fold purification
Crude enzyme	1950	100	195390	2.2	4327	45	100
Ultra filtration (50 kD) Retentate	160	1203	192483	3.14	502	383	851
60% (NH_4_)_2_SO_4_ fraction	17.5	3179	55638	2.7	47	1184	2631
HIC fraction	40	314	12560	0.085	3.4	3694	8208
